# Venetoclax plus azacitidine in higher-risk myelodysplastic syndromes: a treatment-purpose framework for interpreting response depth

**DOI:** 10.3389/fmed.2026.1913068

**Published:** 2026-08-24

**Authors:** Xinfu Zou, Jinyang Bu, Aojiao Chu, Ling Shi, Zheng Zhang, Qin Zheng, Meihong Luo

**Affiliations:** Baoshan Hospital, Shanghai University of Traditional Chinese Medicine, Shanghai, China

**Keywords:** allogeneic hematopoietic stem cell transplantation (aHSCT), azacitidine, higher-risk myelodysplastic syndromes, treatment-purpose stratification, venetoclax

## Abstract

Venetoclax plus azacitidine has demonstrated substantial clinical activity in treatment-naïve higher-risk myelodysplastic syndromes (HR-MDS), including high rates of complete remission, marrow complete remission, and overall response. However, recent phase 3 experience indicates that greater response depth does not necessarily translate into improved overall survival. We propose a treatment-purpose framework for interpreting the clinical value of venetoclax plus azacitidine in HR-MDS. This framework first distinguishes its potential use as a bridge to allogeneic haematopoietic stem cell transplantation from its use for non-transplant disease control. For patients treated with transplant intent, rapid blast reduction, preservation of transplant readiness, the proportion proceeding to transplantation, and post-transplant outcomes should be prioritised. For patients without transplant intent, durable haematological improvement, transfusion independence, symptom control, infection burden, hospitalization, treatment interruption, and quality of survival may be more clinically meaningful. Molecular and cytogenetic variables, including TP53 status, complex karyotype, and IPSS-M risk, should currently support stratified evaluation rather than direct treatment selection. Future studies should align response assessment and clinical endpoints with treatment intent, molecular risk, tolerability, and long-term outcomes.

## 1 Introduction

Higher-risk myelodysplastic syndromes (HR-MDS) remain therapeutically challenging, with limited non-transplant options and poor survival despite the established role of azacitidine (1–3). The phase 1b study by Garcia et al. (4) provides important prospective evidence that venetoclax plus azacitidine is clinically active in treatment-naïve HR-MDS. At the recommended phase 2 dose, complete remission (CR), marrow CR (mCR), and modified overall response were achieved in 29.9, 50.5, and 80.4% of patients, respectively, with a median overall survival (OS) of 26.0 months (4). These results are encouraging, particularly in a disease setting in which improvement over hypomethylating-agent monotherapy has been difficult to achieve.

However, recent phase 3 experience with hypomethylating-agent-based combinations, including venetoclax-containing strategies, has underscored that higher response rates do not necessarily translate into improved OS in HR-MDS (5). This distinction is especially relevant in HR-MDS, where marrow response, hematological improvement, transfusion independence, infection risk, treatment interruption, transplant eligibility, and quality of survival all contribute to clinical value (6, 7). The available data therefore suggest that venetoclax plus azacitidine should not be viewed simply as a universal replacement for azacitidine, but rather as a regimen whose value depends on treatment purpose and patient context.

In this Perspective, we propose a hierarchical treatment-purpose framework that first separates potential bridging to allogeneic hematopoietic stem cell transplantation from non-transplant disease control and then incorporates molecular risk, clinical characteristics, response kinetics, and tolerability as cross-cutting stratification variables (Table 1).

**Table 1:** Treatment-purpose framework and cross-cutting stratification factors for venetoclax plus azacitidine in higher-risk myelodysplastic syndromes.

Treatment intent	Suitable population	Cross-cutting stratification factors	Principal value, risks, and priority endpoints
Transplantation-bridging strategy	Transplant-eligible patients with HR-MDS, particularly those with excess blasts or a need for rapid cytoreduction before allogeneic hematopoietic stem cell transplantation.	Age and fitness; comorbidities; donor availability; baseline cytopenias and infection risk; disease burden and kinetics; TP53 status/allelic state; complex karyotype; IPSS-M category; early response; count recovery; tolerability.	Value: rapid blast reduction and preservation of transplant readiness.
			Risks: infection, prolonged cytopenia, treatment interruption, and delayed transplantation.
			Priority endpoints: time to response; blast reduction; pre-transplant disease status; proportion transplanted; time to transplantation; reasons for failure to proceed; relapse; non-relapse mortality; and post-transplant overall survival.
Non-transplant disease control	Transplant-ineligible patients with HR-MDS who are considered suitable for disease-directed therapy after comprehensive assessment of performance status, frailty, comorbidities, baseline cytopenias, infection risk, and molecular risk profile.	Performance status; frailty and comorbidity burden; baseline cytopenias and transfusion dependence; infection and hospitalization risk; molecular/cytogenetic risk; early toxicity; count recovery; dose intensity; treatment interruptions.	Value: durable hematological improvement, transfusion and symptom benefit, and disease control.
			Risks: myelosuppression, infection, hospitalization, and treatment interruption.
			Priority endpoints: duration of response/hematological improvement; transfusion independence; patient-reported symptoms and quality of life; duration of grade ≥3 cytopenia; infection-related mortality; hospitalization days; treatment-free intervals; overall survival; and net clinical benefit.
Transplantation-bridging strategy	Transplant-eligible patients with HR-MDS, particularly those with excess blasts or a need for rapid cytoreduction before allogeneic hematopoietic stem cell transplantation.	Age and fitness; comorbidities; donor availability; baseline cytopenias and infection risk; disease burden and kinetics; TP53 status/allelic state; complex karyotype; IPSS-M category; early response; count recovery; tolerability.	Value: rapid blast reduction and preservation of transplant readiness.
			Risks: infection, prolonged cytopenia, treatment interruption, and delayed transplantation.
			Priority endpoints: time to response; blast reduction; pre-transplant disease status; proportion transplanted; time to transplantation; reasons for failure to proceed; relapse; non-relapse mortality; and post-transplant overall survival.

## 2 A treatment-purpose framework for venetoclax plus azacitidine

The proposed framework distinguishes two principal treatment purposes: transplantation bridging and non-transplant disease control. These settings differ in their immediate therapeutic objectives, acceptable risks, and priority endpoints. Molecular and cytogenetic risk, baseline clinical fitness, early response, count recovery, and tolerability should be evaluated across both treatment-purpose groups rather than treated as independent indications for therapy.

### 2.1 Venetoclax plus azacitidine as a potential bridge to transplantation

The first treatment purpose is potential bridging to allogeneic hematopoietic stem cell transplantation. In the Garcia et al. (4) cohort, 42 of 107 patients (39.3%) proceeded to transplantation as their immediate next treatment after a median of 4.8 months (range, 1.4–16.8), and 46 of 107 patients (43.0%) underwent transplantation overall. Among the 42 patients proceeding directly to transplantation, the best documented responses before transplantation were CR in 16 patients (38.1%), mCR in 22 (52.4%), and stable disease in 4 (9.5%) (4).

These findings suggest that the regimen can produce disease responses compatible with proceeding to transplantation, but they do not establish that venetoclax plus azacitidine itself increases transplantation rates or improves post-transplant outcomes. Only 34 of 107 patients (31.8%) were considered transplant-eligible at study entry, whereas 20 patients initially considered ineligible ultimately underwent transplantation, including 17 immediately after study treatment (4). This discordance underscores that transplant eligibility was dynamic and that the transplanted subgroup was selected according to age, performance status, donor availability, treatment response, and other clinical considerations.

The study did not report patient-level reasons for failure to proceed to transplantation, post-transplant relapse, or non-relapse mortality. Accordingly, the available findings support further evaluation of venetoclax plus azacitidine as a potential bridging strategy. Prospective comparative studies should report the proportion proceeding to transplantation, time to transplantation, reasons for failure to proceed, disease status before transplantation, post-transplant relapse, non-relapse mortality, and post-transplant survival. Outcomes in patients treated with transplant intent should also be reported separately from those treated with non-transplant intent, because survival after study treatment may be strongly influenced by transplantation and associated selection effects.

Therefore, venetoclax plus azacitidine should currently be regarded as a potential transplantation-bridging strategy rather than a proven intervention for increasing transplantation rates or improving post-transplant survival.

### 2.2 Venetoclax plus azacitidine for non-transplant disease control

The second treatment purpose is disease control in transplant-ineligible patients who are considered suitable for disease-directed therapy after comprehensive assessment. In this setting, the principal objective is not rapid cytoreduction alone, but sustained disease control with acceptable toxicity and preservation of quality of survival.

This population is clinically heterogeneous, encompassing substantial differences in performance status, frailty and comorbidity burden, baseline cytopenias, transfusion dependence, infection risk, hospitalization burden, and molecular risk profile, and therefore should not be regarded as a single therapeutic group. The phase 1b study enrolled patients with an Eastern Cooperative Oncology Group performance status of 0–2, and only 7 of 107 patients (6.6%) had a performance status of 2; its findings may therefore not generalize to substantially frail patients or those with poorer performance status (4).

Given that the phase 3 VERONA trial did not demonstrate an OS benefit, it remains unclear which clinically defined non-transplant subgroups derive meaningful benefit from the regimen (5). For patients who are not transplant candidates, prolonged myelosuppression, infection-related morbidity, hospitalization, and treatment interruption may offset gains in marrow response. Accordingly, the non-transplant category should be regarded as a proposed framework for patient stratification and outcome evaluation based on current evidence, rather than as an established treatment-selection standard.

Priority outcomes in this group should include duration of response and hematological improvement, durable transfusion independence, patient-reported symptoms, quality of life, infection-related mortality, hospitalization days, treatment-free intervals, and overall net clinical benefit.

## 3 Cross-cutting stratification factors

### 3.1 Molecular and cytogenetic risk

Molecular and cytogenetic risk should be incorporated across both treatment-purpose groups rather than treated as a third category parallel to treatment intent. The Molecular International Prognostic Scoring System (IPSS-M) and the molecular emphasis of recent World Health Organization and International Consensus Classification frameworks have changed the way MDS is risk-stratified (8–10).

In the Garcia et al. (4) study, patients with TP53-mutated disease had numerically shorter survival than the overall population, and IPSS-M data were not available. TP53 alteration, complex karyotype, and high-risk IPSS-M categories are established prognostic variables, but current evidence is insufficient to show that they predict differential benefit from venetoclax plus azacitidine. In particular, there is no validated basis for selecting treatment according to an individual TP53 mutation site; TP53 allelic state and mutation burden also remain prognostic rather than validated predictive factors in this setting.

Their present value is therefore to support pre-defined molecularly and cytogenetically stratified analyses, not to direct treatment selection. Establishing predictive value will require comparator-based studies with pre-specified treatment-by-biomarker interaction analyses rather than outcome comparisons within a single treatment cohort alone. Molecular variables should therefore be used to define analytical strata and generate hypotheses regarding treatment heterogeneity, but not as stand-alone criteria for selecting venetoclax plus azacitidine.

### 3.2 Response rate, depth, speed, and duration

A central limitation of current evidence is the tendency to summarize treatment activity using a single overall response rate. Response evaluation should instead distinguish response rate, speed, depth, and duration.

For patients being prepared for transplantation, time to response and rapid reduction in marrow blast percentage may be clinically important because prolonged cytopenia or delayed response can jeopardize transplant timing. For patients treated without transplant intent, sustained hematological improvement, durable transfusion independence, symptom control, quality of survival, and time without hospitalization or treatment may be more relevant than a transient marrow response.

Traditional International Working Group (IWG) 2006 criteria have been widely used, but their limitations in HR-MDS are increasingly recognized (6, 7). Marrow CR, even with hematological improvement, is biologically meaningful but should not be interpreted as equivalent to CR, durable disease modification, or improved survival. Response rate is an important measure of treatment activity, but it should not be used as the sole basis for assessing long-term benefit or net clinical benefit.

### 3.3 Tolerability and net clinical benefit

The clinical value of venetoclax plus azacitidine cannot be assessed independently of treatment exposure and tolerability. In the phase 1b study, 94.4% of patients experienced grade 3/4 treatment-emergent adverse events, 68.2% experienced serious adverse events, and 77.6% experienced grade 3/4 neutropenic events; venetoclax dose reductions occurred in 52.3%, treatment interruptions in 89.7%, and the median dose intensity was 76.9% (4).

Prolonged neutropenia, thrombocytopenia, infection, hospitalization, dose reduction, and treatment interruption may reduce or even offset the value of deeper marrow responses. This consideration is particularly important in older or transplant-ineligible patients, for whom preservation of functional status and avoidance of prolonged hospitalization may be major therapeutic priorities.

Future studies should therefore report time to count recovery, cumulative duration of grade ≥3 neutropenia and thrombocytopenia, infection density, antimicrobial exposure, hospitalization days, transfusion burden, relative dose intensity, treatment-free intervals, and reasons for dose reduction, interruption, or discontinuation. These measures should be integrated with survival and response outcomes to define net clinical benefit.

## 4 Discussion and future directions

Venetoclax plus azacitidine remains a promising and biologically plausible strategy in HR-MDS. The negative OS result of the VERONA trial should not be interpreted as evidence that the regimen has no role. Rather, it suggests that its role is unlikely to be broad and unselected and that response depth alone is insufficient to establish long-term clinical benefit.

Our proposed framework positions treatment intent as the first level of stratification. Transplantation bridging and non-transplant disease control represent clinically distinct settings and require different definitions of benefit, risk, and relevant endpoints. Molecular and cytogenetic risk, early response kinetics, count recovery, and tolerability should then be evaluated as cross-cutting factors within each treatment-purpose group.

The available evidence suggests that venetoclax plus azacitidine may represent a feasible pre-transplant strategy for selected patients with HR-MDS. However, prospective comparative studies are required to determine whether this approach increases transplantation rates, improves post-transplant outcomes, or provides advantages over existing treatment strategies. In the non-transplant setting, studies should determine whether deeper responses translate into durable hematological benefit, reduced transfusion burden, improved symptoms, fewer hospitalizations, and better quality of survival.

Future prospective trials and real-world analyses should stratify patients according to treatment intent at study entry, report transplant-specific outcomes separately, incorporate pre-defined molecular and cytogenetic subgroups, and evaluate response kinetics together with tolerability and longitudinal clinical benefit. Comparator-based biomarker interaction analyses will be required before molecular features can be used for treatment selection. Studies should also define early response-adapted venetoclax duration and pre-specified infection-prevention and dose-modification pathways.

In HR-MDS, the value of response depth can only be understood in the context of treatment purpose, molecular risk, tolerability, and long-term patient benefit.
